# The opioid receptor: emergence through millennia of pharmaceutical sciences

**DOI:** 10.3389/fpain.2023.960389

**Published:** 2023-11-01

**Authors:** Carolyn A. Fairbanks, Cristina D. Peterson

**Affiliations:** ^1^ Department of Pharmaceutics, University of Minnesota, Minneapolis, MN, United States; ^2^ Department of Pharmacology, University of Minnesota, Minneapolis, MN, United States; ^3^ Department of Neuroscience, University of Minnesota, Minneapolis, MN, United States

**Keywords:** opioid receptor, history, opium, morphine, naloxone, endorphins

## Abstract

Throughout history humanity has searched for an optimal approach to the use of opioids that maximizes analgesia while minimizing side effects. This review reflects upon the conceptualization of the opioid receptor and the critical role that the pharmaceutical sciences played in its revelation. Opium-containing formulations have been delivered by various routes of administration for analgesia and other therapeutic indications for millennia. The concept of a distinct site of opium action evolved as practitioners developed innovative delivery methods, such as intravenous administration, to improve therapeutic outcomes. The introduction of morphine and synthetic opioids engendered the prevalent assumption of a common opioid receptor. Through consideration of structure-activity relationships, spatial geometry, and pharmacological differences of known ligands, the idea of multiple opioid receptors emerged. By accessing the high-affinity property of naloxone, the opioid receptor was identified in central and peripheral nervous system tissue. The endogenous opioid neuropeptides were subsequently discovered. Application of mu-, delta-, and kappa- opioid receptor-selective ligands facilitated the pharmacological characterization and distinctions between the three receptors, which were later cloned and sequenced. Opioid receptor signal transduction pathways were described and attributed to specific physiological outcomes. The crystal structures of mu, delta, kappa, and nociceptin/orphanin FQ receptors bound to receptor-selective ligands have been elucidated. Comparison of these structures reveal locations of ligand binding and engagement of signal transduction pathways. Expanding knowledge regarding the structure and actions of the opioid receptor fuels contemporary strategies for driving the activity of opioid receptors toward maximizing therapeutic and minimizing adverse outcomes.

## Introduction

In 2021 and 2022, over one hundred and sixty thousand people lost their lives to the opioid epidemic in the United States (1) leaving behind heartbroken family members, devastated friends, and grieving communities. The chronic pain crisis is a separate but related ongoing public health problem with an intersection of individuals that suffer and struggle between these two neurological conditions. Analgesic options are frequently inadequate leaving those with the most severe pain the only option of opioid therapy, which carries the very serious risk of addiction among other adverse side effects and social stigma. This struggle with seeking adequate pain relief while managing and/or minimizing risk of addiction is an ongoing duality with which humanity has struggled throughout recorded history. Through considering how we as a human society have approached a global problem, we can understand better how we have come to a current state of the same problem. This reflection may yield insights as to how the ongoing opioid epidemic may be most effectively addressed in the present day. This review does not represent a comprehensive survey of the extensive chemical, pharmacological, physiological, and molecular biological literature on mu, delta, kappa, or nociceptin/OFQ receptors or on the opioid receptor epidemics. Excellent reviews on those topics have been contributed elsewhere and some have intentionally been cited within this piece. Rather, this review seeks to reflect upon how the concept of a site of action (receptor) for opium and later morphine emerged, particularly through the pharmaceutical sciences.

Recognition of the pain-relieving effects of *Papaver somniferum*, or the Red Poppy from which opium is extracted, goes back millennia. While the therapeutic effects of opium were readily appreciated historically, so were the deleterious effects. The drive to find an optimal analgesic compound based on opium that does not cause addiction has fueled the efforts many healers, doctors, chemists, pharmacologists, molecular biologists, regulators, funders, and sponsors for thousands of years. How to create an opium formulation or a new opioid compound that has all the benefits of analgesia without sedation or addiction is a reverberating question throughout the history of healing, medicine, and science. Understanding where and how opium/morphine acts upon the body to exert these effects has always been considered key to that search. As several thousand years of opium use unfolded, so the concept of a “site of action” of opium (and later morphine) began to emerge. The idea of a cell site or a receptor site took shape perhaps as early as the 1600s when practitioners using opium to treat chronic pain began to innovate in methods to deliver opium formulations closer to the site of action. By the mid 1800s, practitioners experimenting with direct injections began to engage in the debate as to the site of action of opium, specifically whether injected opioids act locally or systemically. The early 1900s saw the development of opioid analog antagonists that reversed the action of opioid agonists; by the mid-century, the understanding of the stereospecificity of the opioids accelerated acceptance of the concept of a common opioid receptor site. In the early 1970s, through using the high affinity attributes of the opioid antagonist naloxone, the existence of the opioid receptor was revealed. By the 1990s, the amino acid sequences of all three opioid receptors were established. Through molecular manipulation of the opioid receptors, a greater understanding of how biologically activated opioid receptors exert their therapeutic and adverse effects has been gained. Pursuit of how to maximize the therapeutic effects and minimize the adverse effects now centers around how to drive opioid receptors or combinations of receptors toward that goal. Through this article, we review the development and persistence of the initial formulations of opium, the beginning of alternative forms of percutaneous drug delivery, the monumental impact of medicinal chemistry on the extraction of morphine, and introduction of the antagonists. All these stages in the history of the use of opium greatly aided the emergence of the concept of the opioid receptor and ultimately the discovery of the opioid receptor in the nervous system. Note how the goal of finding a better analgesic through innovation is a common theme throughout nearly all of time.

## Botanical period

Evidence of early human recognition of a biological response to parts of the *Papaver somniferum* is offered in the form of both written uses for medicinal purposes and artistic renderings of poppy flowers, sometime in association with various goddesses of harvest and of healing (2). Throughout this time, opium was recognized as a routine constituent of botanical medical mixtures. Between 132 and 62 BCE, Crateuas, physician to King Mithridates the VI of Pontus, composed a 40 ingredient antidote that was originally intended to protect the monarch from poisoning (3). The Mithridatium, as the formulation was called, contained 40 ingredients, including toxins from various snakes, scorpions, and sea-slugs, but also opium. Following Mithridates' military defeat by Pompey and his death, the composition was discovered, preserved, translated and communicated to the world (3).

Andromachus the Elder of Crete was employed by the emperor Nero as his physician in the first century. Crete was known for botanical expertise and herbal exports. Educated in this herbal knowledge, Andromachus offered effective herbal remedies including the *Theriac Andromachus*, a 64 constituent formulation intended to be an improvement over the Mithridatium with notably a perceived “much greater quantity of opium” (4). Andromachus is said to have enshrined the formulation [entitled Galene or “Tranquility” (4, 5)] into a Greek poem (5) which was revised and used in botanical medicine for 2000 subsequent years and came to be thought of as an effective treatment for many ailments and diseases beyond the original intent as an antidote to poisoning. The Greek pantheon includes the Goddess of the cure, Panacea (Panakeia), a name that became synonymous with a universal remedy (3). Thus the Theriac Andromachus was thought by many to be a panacea for many ailments for many centuries (e.g., malaria and plague (4), neurological disorders and organ failure (5)).

The historically famous Greek physician Galen, court physician of Marcus Aurelius (5) and Lucius Verus, also expanded the formulation Theriac Andromachus to 70 ingredients to be delivered either orally, ocularly, or topically (3, 4) representing, perhaps, the first documented intentional transdermal delivery of an opioid therapeutic. In subsequent centuries a definitive diaspora of variations of Theriac Andromachi was consumed by monarchs (e.g., King Alfred the Great (4) and Queen Elizabeth I (5)) and communities [Germany, Italy (5), and France] alike, often with broad public acceptance, support, and celebration (6). The effectiveness of mithridatium or theriac was broadly viewed to be dependent on adherence to what was considered the proper protocols of manufacturing. In other words, if a batch did not provide the intended therapeutic outcome, the explanation given was insufficient quality control (5). The manufacturing of theriac therefore was thought to be best performed publicly, and, as such, became the focus of national annual festivals in Germany, and France and Italy (5). Some of the ingredients were difficult to come by (5), the process took multiple days to prepare (e.g., 40) and a varying number of years (up to 12) were needed to allow it to mature (4). Public displays of manufacturing evolved to the development of regulatory inspections of facilities and final material (4). The word “theriac” derives from the Greek word “theria” refers to “wild beast” (3) but later evolved in translations somehow to became recognized as “treacle”, a term more commonly associated with molasses in modern times. It may be that sweeteners were added to the formulation as part of the pharmaceutical preparations contributing to the evolution of the term (5). “Venetian Treacle” was an Italian form of theriac that was widely exported (4).

In 1745, nearly 2000 years after the introduction of theriac, English physician William Herberden published an impactful and definitive critique (7) of the persistent and continuing use of theriac as contradictory to the state of scientific and medical knowledge of his time. He asserted that the inclusion of the number of competing ingredients, the proportions of ingredients, and the combination lacked clear rationale. He particularly called out concern with the risks of opium in the mixture. Herberden writes:

 “*As many people busy themselves with the practice of Physic, who are unqualified to know what they are doing, it may be advisable, for the sake of such as fall into their hands, to discountenance a medicine, which, upon the tradition of sovereign virtues, or as a sudorific, is often applied at random, and, by means of the Opium, does much mischief. But its* *use may be of ill consequence not only in the hands of the vulgar, but even of a skillful Physician; for Opium or any powerful drug, mixed up into an electuary with so many other things, is against all rules of pharmacy; the prescriber lies too much at the mercy of the person who mixes the ingredients, whether what he gives for an ordinary dose shall not contain a dangerous or fatal quantity of opium: and indeed it is hardly to be expected, in such a multiplicity of ingredients, that the usual dose will contain a just proportion of all of them, and of course, the Physician will be greatly in the dark whenever he prescribes it”* [p. 14–15, Heberden, 1745 (*7*)].

This critique had significant impact and heralded the decline of the use of theriac globally (6). Mithridatium and Therica Andromachus (Galene) appear for the last time in the *London Pharmacopoeia* in 1746, in the *German Pharmacopoeia* in 1872, and the *French Pharmacopoeia* 1884 (4). The *London Pharmacopoeia* of 1788 explicitly repudiated the use of theriac; the French Codex of 1908 noted definitive removal of theriac from approved use (6), noticeably approximately 2000 years after the product was introduced.

The first intravenous injection of opium is attributed to the English architect of St. Paul's Cathedral, Sir Christopher Wren (1632–1723), who used a goose quill to inject opium solution into a dog vein using an animal bladder as the reservoir (8). He reported that the dog was sedated. The pharmacist Friedrich Sertürner isolated morphine from opium in the early 1800s, which represented the first time an alkaloid was isolated from a plant product (9), an event of noted significance in chemistry. He tested the new compound in rats, mice, cats, and dogs (10, 11) and observed the effects to be similar to that of opium. It is said that when Serturner was suffering from a toothache that he took his purified product and noted the alleviation of his own pain (9). Through experimentation on himself and three young male human research participants (11) he identified dose-effect relationships. He observed that 30 mg of the drug induced a light euphoria, 60 mg of the drug induced fatigue, and 90 mg resulted confusion and sedation (9).

Shortly after Serterner's extraction, morphine was manufactured and available for oral delivery. An advantage of the extraction and purification of morphine was the ability of practitioners to offer patients morphine for pain in predictable doses, greatly reducing the risk of overdose from non-standardized opium tinctures (9). However, significant prevalence of use did not expand until the introduction of the hypodermic needle. In 1836, the French physician G.V. Lafargue adopted a vaccination approach to introduce a morphine paste subcutaneously. He dipped the point of a vaccination lancet in morphine and then introduced it under the skin, horizontally, for a few seconds (10); he reportedly performed the procedure repeatedly to his own forearm and noticed himself becoming sedated (8). The first medical reports of the use of opium for controlling post-operative pain was reported using oral laudanum formulations in the late 1700s and early 1800s; opium rectal suppository was introduced as a post-operative medication in 1891 (8). The concept of local vs. systemic action of opioid was, in fact, already under consideration and debate in the mid 1800s. In 1853, the hypodermic needle was introduced via application of a syringe and hollow needle to deliver morphine directly into a painful area by Dr. Alexander Wood to a female patient suffering neuralgia (10). The intent was to anesthetize the peripheral nerves at the local level (10), but the patient also experienced significant sedation (8) which would suggest a systemic action. A second individual, Charles Hunter, tried to replicate the strategy in 1) a painful site and 2) a site distal from the painful site. He noted delivery of morphine to both sites gave equal alleviation of pain. From this, he deduced that the effect of morphine was due to systemic action of opioid whereas Dr. Wood continued to assert a local action. This difference in interpretation escalated into a dispute in the medical literature that included an argument regarding who should receive credit for introducing the technique. Ultimately the community gave credit to Wood for introducing the technique and Hunter for establishing a systemic action needle (10). Another example of early recognition of the potential benefit of direct delivery to the presumptive site of opioid action was the first local spinal injections (12), initially of cocaine, in 1885 by Leonard Corning. In 1899–1901, morphine mixed with cocaine or other local anesthetic was delivered spinally by physicians on three separate continents including Dr. Matas of New Orleans (13), Dr. N. Racoviceanu-Pitesti of Romania (14), and Dr. Otojiro Kitagawa of Japan (15). The spinal site of action would remain largely underexplored until the report of morphine delivery via chronic catheterization of rat by Rudy and Yaksh in 1976 (16).

Dr. Wood went on the optimize the hypodermic method by graduating the barrel of the syringe and reducing the size of the needle (10). Repeated hypodermic injections of morphine (up to 1000s of injections in a single patient) to treat neuralgia became commonplace. In 1870, Professor Thomas Clifford Allbutt of the University of Cambridge wrote about his increasing discomfort with his awareness that many doctors were treating patients “who have been injecting themselves daily or more than daily during long periods of time for neuroalgesia which seem as far from the cure as they were at the outset” (10). Of his own patients he noted that “they all find relief in the incessant use of the syringe and they all state that without the syringe life would be insupportable” (10). The dual advantage of morphine's increased potency relative to opium and the direct access to the systemic circulation to provide faster distribution to the active site (the opioid receptors as yet unidentified) further drove the increase in morphine use, particularly for use in the military during warfare (11). Appreciation of the risks of addiction and of infection were delayed for many years but were generally understood by 1885 (10). In the late 1800s and the early 1900s, the United States and other countries began the establishment of legislation and agreements to restrict the use, manufacture, imports, and sales of opium (11).

## Medicinal chemistry

Although Friedrich Sertürner is credited with the first publication of the isolation of morphine, it has been proposed that several other chemists and pharmacists extracted substances from opium presumed to be the active compound as early as the mid-1600s (11). Following the isolation of morphine, the next significant steps were elemental analysis and identification of the chemical formula of morphine by the French chemist Auguste Laurent in 1847 (11) followed by establishment of the structural formula by Sir Robert Robinson in 1925, an accomplishment that contributed to his later receipt of the Nobel Prize in Chemistry in 1947 (11). Note the greater than 100 years that spanned the time between the isolation of morphine from opium to establishing the structure. In 1956, Gates and Tschudi confirmed Robinson's structure through the first synthesis of morphine (17).

Of similar significance in the realm of medicinal chemistry was the synthesis and commercialization of diacetylmorphine, later called heroin. Diacetylmorphine was synthesized (along with acetylcodeine and acetylmorphine) by the English chemist Charles Romley Alder Wright in 1874 (18). This event is broadly viewed as an effort to synthesize a new opioid compound with less addiction propensity than morphine representing another point in history where the universal goal to seek a better analgesic was pursued. Alder Wright enlisted a colleague, Dr. F.M. Pierce, MD, to assess these acetylated opioids in dog and rabbit, but the results were not interpretable since morphine was not included as a positive control (19). In 1888, a group at the University of Edinburgh conducted studies of impact of dose on the effects of morphine and a series of derivatives, including diacetylmorphine, in frog and rabbit pharmacological assays of tetanus and depression (20); they observed that diacetylmorphine had a greater effect than morphine on depressing spinal cord and respiratory centers in these assays (19) although these observations may not have been broadly appreciated.

In the early 1890s, the head of Bayer's Pharmaceutical science laboratory, Arthur Eichengrün and his assistant Felix Hoffmann, set about acetylating a variety of compounds such as tannins, salicylic acid, and morphine (19). Bayer was reportedly interested to identify an improved antitussive medication to control coughing particularly in patients with respiratory disease with limited side effects, a common objective in opioid development. Following a series of pre-clinical experiments and clinical trials, the head of the Bayer laboratory of Pharmacology, Heinrich Dreser, reportedly observed that diacetylmorphine suppressed cough and stimulated respiration and seemed to have little respiratory depressant effects (18), the latter of which was incongruent with standard opioid pharmacology. Although they could not claim the patent due to the prior art in the publication of Alder Wright, Bayer made the decision to manufacture and market diacetylmorphine staring in 1898 as a cough suppressant under the name “Heroin” which is presumed to be associated with the word Heroish, referring to “powerful” and “extreme” (19). As heroin and other opioids were marketed intensely and broadly distributed, the incidence of heroin abuse escalated rapidly.

During the last third of the 19th century, regulation of the medical use and pharmacological production in the United States was the domain of the individual states; many states reportedly elected to refrain from establishing controls over opium and opiate manufacture and medical use (21). Consequently, opium and opiates were broadly available, intensely marketed, and widely used. Crude opium imports to the United States escalated from 1870 to 1895 (21). By the early 1900s, legislation was enacted to restrict (1914, Harrison Act, U.S. Congress) and then later ban (1924, USA) the medical use of heroin (19). New legal restrictions resulted in a decline in crude opioid imports from 1900 to 1910 (21).

It is also noteworthy that the medical heroin experience was accompanied by introduction of a variety of other morphine analogs including hydrocodone (a component of Vicodin) and hydromorphone (Dilaudid) (18), important medicines in widespread use for control of pain in the present day (18).

Following the conclusion of World War II, the Allied Powers acquired access to the records of the infamous (22) German industrial chemical conglomerate I.G. Farbenindustrie (23). The United States State Department sent members of the *Technical Industrial Intelligence Committee* to evaluate their research activities. During this process, the chemical composition known as Hoecht 10820 (6-dimethylamino-4,4-diphenyl-3-heptanone) was identified as of therapeutic interest. I.G. Farbenindustrie scientists had synthesized in 1938 (24) and developed it under the name amidon (23), or polamidon or palamidon (24). By 1948 6-dimethylamino-4,4-diphenyl-3-heptanone was assigned the generic name of methadon which ultimately became methadone. Eli Lilly acquired the patent (for $1) and marketed it as Dolophine. Methadone (together with buprenorphine) is now listed among the WHO's 100 essential medicines for its effectiveness in addiction treatment due to its long half-life (25). It is also recognized for curbing the spread of HIV due to reduced behaviors associated with HIV transmission (25, 26). In addition to methadone, pethidine (meperidine or Demerol) was also initially synthesized by I.G. Farbenindustries in the late 1930s (24).

Another significant phase in opioid chemical research was a comprehensive study of structure-activity relationships. This effort was initiated by the United States' Committee on Drug Addiction of the National Research Council (1929–1939) and then assumed by the National Institutes of Health (18). Here again the objective was to understand the impact of structural modification on the pharmacology to identify or design an optimal opioid medication with reduced side effects, in other words, to find a better analgesic. Over 150 morphine derivatives and 300 synthetic compounds were studied at the University of Michigan for their standard opioid pharmacological effects. Although several important contributions emerged from this program (e.g., oxycodone and oxymorphone), identification of a strategy to maximize the therapeutic effects over adverse effects remained elusive (18).

Synthesis of the highly potent oripavine series represents the next notable development, occurring in the 1960s. This series includes etorphine, used in large animal veterinary practicr to sedate large mammals such as elephants (27), as well as the partial agonist buprenorphine, which has become an important treatment option for opioid use disorder. In 1963, Janssen (28) reported the development of the 4-anilidopiperidine class of opioid agonists and introduced fentanyl as a opioid with significantly greater potency relative to morphine. Highly lipophilic, fentanyl is effectively delivered by nearly every route of administration and has been used intravenously as an anesthetic and to treat post-operative pain, oral/transmucosally (e.g., fentanyl lollipop) to treat cancer pain, and transdermally (e.g., fentanyl patch) to treat chronic severe pain in opioid tolerant patients as well as other transmucosal immediate release fentanyl preparations such as oral/buccal sublingual and nasomucosal delivery strategies (29). Broad availability has also rendered the dosage forms abusable via a variety of routes of administration. Fentanyl is attributable to a significant fraction of the current opioid epidemic, amplified notably by illicit manufacture (29, 30). In recent years, the ultra-potent potent analog carfentanil, which has historically also been used in large mammalian veterinary practice and wildlife herd management, has increased in illicit use and contributed to opioid overdoses (31), as has a broad array of fentanyl analogs that are manufactured illicitly and trafficked into the United States (30).

## Receptor revelation through stereoselective pharmacology

The idea that opioids bind to a macromolecular protein receptor that resides in or on the surface of a neuron (32) emerged during the mid-20th century and was based in large part upon the synthesis and introduction of a number of opioid derivatives that opposed the actions of morphine, what we now know as the opioid antagonists.

*N-Allyl- opioids:* The first demonstration of antagonism of morphine by another opioid is uniformly attributed to the work of a German chemist, J. Pohl, published in 1915 (12, 18). Pohl was reportedly interested in the observation of a colleague who had decided that allyl compounds stimulated the respiratory system. Pohl theorized that by adding an allyl group to opioid alkaloids, the effects of opioids on the nervous system may be countered (12). He then observed that his synthetic product of N-allylnorcodeine reversed the respiratory depression induced by morphine in rabbit and dog (12, 18). This monumental observation of millennial importance heralded the introduction of a potential antidote for the lethal effects of opium, a key constituent of a mixture (Mithridatium) that was originally intended to serve as an antidote to poison itself. Given the significance of such a step forward, it is surprising that a period of 25 years would elapse before the next step was taken. In the early 1940s, N-allyl-Oallyl-normorphine and N-allylnormorphine (nalorphine) were synthesized and demonstrated to reverse respiratory depression induced by morphine as well as other opioid agonists in both animal subjects and humans. The ensuing pharmacological studies of the effects of nalorphine and nalorphine + diverse opioid agonists resulted in complex outcomes thoroughly reviewed contemporaneously by the renowned clinical pharmacologist Dr. Louis Lasagna. To summarize the initial observations, it was noteworthy that nalorphine, when given at morphine equivalent doses, did not induce classic signs of opioid side effects such as vomiting, sedation, restlessness, and Straub tail but at higher doses did have comparable LD50 values. In humans, a variety of aversive or dysphoric side effects were noted with N-allylnormorphine when given alone, but sedation induced by morphine or other opioids was often reversed. Analgesia was evident at high doses. In considering the mechanism for how nalorphine reversed the effects of opioids, the theory of competitive antagonism at specific “*cell sites”* (12) or *receptors* (33) was advanced. It was also recognized that such a mechanism did not fully explain the complex pharmacology exhibited by nalorphine (12), such as the findings of Houde and Wallenstein that nalorphine antagonized morphine analgesia at lower doses to a greater extent than at higher doses (34). Throughout the ensuing twenty-five years, multiple N-Allyl-opioid antagonist (nalorphine, levallorphan) pharmacological experiments were performed using a variety of opioid agonists (e.g., morphine, methadone, levorphan, dihydrocodine, phenazocine among others) observing dependent measures (thermal reflex, intestinal spasm, lenticular opacity); this literature was efficiently summarized by Grumbach and Chernov (33). The parallelism of the rightward shifts observed in morphine dose-response curves in the presence of nalorphine by multiple investigators (35–38) in diverse species with distinct dependent measures combined with their own internally controlled comparison using ten distinct opiate agonists offered compelling evidence in support of a common receptor site (33) for opioid agonists (isolated and/or introduced in the 19th century) and the opioid antagonists (synthesized in the 20th century). *Naloxone:* In 1961 N-Allylnoroxymorphine, naloxone, was introduced and reported to antagonize oxymorphone analgesia in mice, respiratory depression in human (39), and later meperidine, alphaprodine as well as oxymorphone in human (40). This new tool would ultimately have a sweeping impact on science and humanity. Naloxone became the primary gold standard to define any opioid ligand and the primary tool to save lives following opioid overdose, following its introduction to the market in 1971 (41). Unlike the opioid agonist-antagonists, naloxone reversed the effects of morphine but had no agonist activity when delivered as a single agent. This distinct pharmacological profile supported the proposal that opioid antagonists competed with agonist directly at a *receptor* and diminished residual views that the antagonists acted by stimulating respiration (34).

*Stereospecificity:* A key feature of mid-twentieth century opioid neurochemistry was the recognition that, in many cases, opioid ligands are chiral and one enantiomer demonstrated significantly greater analgesia (efficacy and/or potency) than the corresponding mirror image species (42). Well-known examples included (-) methadone, (-) morphine, and a variety of related compounds whereby the D (-) configurations were active and the L (+) configurations were often inactive (32, 42). It was noted that since the physicochemical characteristics of enantiomers were identical, this diminished the likelihood of biopharmaceutical or pharmacokinetic explanations for the differences in effects. Any differences in effect would most likely be attributable to differences in spatial geometry and corresponding receptor interactions (42). Differences in effect were expected to be due to affinity or intrinsic activity with the presumptive receptor, as yet uncharacterized. It was recognized that stereospecificity, such as was observed with the opioid receptor ligands, typically was associated with interaction with proteins such as receptors (32). The summation of all the medicinal chemistry research from Pohl (1915) through Portoghese (1966) (42) focused on an assumed receptor protein that resides on a neuron and accounts for the pharmacological effects of opioids (32). In his 1952 (43) and 1954 (44) publications, Beckett described the key elements of a potential receptor binding site for morphine. This theoretical receptor surface contained specific aspects that could associate with the common chemical features of opioid ligands (32) These elements were designed from spatial complementarity with key chemical moieties in opioid isomers and from the antagonistic activity of nalorphine. It was suggested that each of the morphine analogs could assume conformations that would interact stereospecifically with a putative receptor that binds morphine. The principle of stereospecificity of opioid drug action was fundamental in the development of a specific receptor for morphine.

## Identification of the opioid receptors

The idea that opioids may engage more than one receptor subtype was initially proposed by Portoghese in 1965–1966 (42, 45). He suggested that multiple receptor species may explain the observed diverse structure-activity features of known opioid molecules that did not seem to fit a simplistic form of drug-receptor interaction, such as binding to the identical site on the same receptor. Shortly thereafter, Martin advanced the concept of receptor dualism (agonists produce the same effect but occupy different receptors) to explain distinct pharmacological effects observed with nalorphine compared to morphine, and that the abstinence syndrome associated with nalorphine and cyclazocine (a mixed opioid agonist/antagonist compound of the benzomorphan class) differed qualitatively from that of morphine abstinence syndrome (34, 46). These diverse responses to morphine, nalorphine, and cyclazocine were observed by Martin in dog. For example, morphine and cyclazocine induced different abstinence syndromes and did not demonstrate analgesic cross-tolerance to one another. Martin and colleagues proposed three different opioid receptors termed mu, kappa, and sigma(34, 47, 48). The initial opioid receptor subtype trio ultimately evolved to include the delta opioid receptor for the distinct pharmacological profile of the enkephalin neuropeptides (49) and to exclude sigma since the sigma receptor does not display several signature features of opioid receptors (50). The experiments reported by Lord and colleagues (51). Comparing the action of morphine, Leu- and Met-Enkephalin, and *β*-endorphin in two distinct tissue preparations (guinea pig ileum and mouse deferens) further supported the existence of mu and delta opioid receptors; the authors also suggested the existence of kappa opioid receptors (51).

It was initially thought that exposure of radiolabeled opioid agonist to brain tissue would reveal the opioid receptor through increased specific binding of the radiolabeled opioid in regions expected to be important in opioid pharmacology. However, such experiments did not demonstrate a selected region with appreciable stereospecific binding of radiolabeled morphine, dihydromorphine, or levorphanol (32). A second pharmacological strategy demonstrated that intraparenchymal delivery of naloxone to the addicted rat induced withdrawal (52) in specific regions. Similarly, delivery of morphine to specific rat brain sites induced analgesia (53) as did delivery to the spinal cord (16). Not surprisingly, these effects were more pronounced when delivered to different regions; however, since the dependent measures were completely different, minimal insight as to where the receptor is more highly expressed could be gained. Improving the portion of binding that was stereospecific came about when high specific activity antagonists, such as [^3^H]naloxone (54) were used as the radiolabeled probe ligand, enabling low concentrations to be applied combined with improved cold washing of homogenates, which removed nonspecific bound ligand (32). In 1973, Candace Pert and Solomon Snyder reported stereospecific [^3^H]binding of naloxone that was exquisitely restricted to both central (brain) and peripheral nervous system tissue (intestine); [^3^H]naloxone was competed by unlabeled naloxone as well as a variety of (-) opioids, but not (+)-opioids nor centrally acting non-opioid ligands such as serotonin, norepinephrine, choline, or histamine among others (54). This report was the clearest and most direct evidence of opioid receptor binding and biochemical evidence of an opioid receptor. Concurrently, Eric Simon and his colleagues evaluated the binding of the high affinity and high potency opioid agonist [^3^H]etorphine which, unlike other radiolabeled agonists of the time, did demonstrate stereospecific binding in rat brain that was competed by naloxone and (-)-levorphanol [but not (+) dextrorphan, the enantiomer of levorphanol] (55). A third contemporary study reported stereospecific binding of [^3^H]dihydromorphine in synaptic plasma membrane fractions of rat brain, binding that was competed by (-)-methadone (but not (+) methadone (56)). Taken together, these three positive findings are largely viewed as the first biochemical evidence for the existence of the opioid receptor. Shortly following the documentation of the existence of the opioid receptor, it was reported that opioids reduced cAMP levels in rat brain homogenate (57) and in neuroblastoma derived cell lines (58). These data established the inhibition of neuronal adenylate cyclase as a key signaling mechanism of opioids. The endogenous opioid receptor ligands were also intensely pursued (59) and the opioid neuropeptides were introduced: enkephalins (60), *β*-endorphin (61, 62), and dynorphin (63). [the endomorphin-1 and −2 neuropeptides were discovered about twenty years later (64)]. The next decade of pharmacological research revealed much about the development of the classic features of opioid tolerance, dependence, and signal transduction. The design, synthesis and introduction of antagonists (65–68) and agonists (69–72) that were selective for mu (67, 69), delta (65, 71, 72), and kappa (66, 68, 70) opioid receptors greatly aided subsequent studies that elucidated the individual pharmacology of each receptor subtype in terms of analgesia, opioid tolerance, cross-tolerance, dependence, signal transduction, and other distinct features of each receptor.

## Molecular biology

Significant strides in molecular biology enabled the pivotal advances in opioid research with the cloning of the mu (73–76), delta (77, 78), and kappa (79–81) opioid receptors in the early 1990s. It is noteworthy that, although prior pharmacological studies suggested multiple subtypes within each mu, delta, and kappa receptor class, only one gene was defined for each (82). Pasternak and colleagues intensely pursued and defined multiple splice variants of the mu opioid receptor that are congruent with observed mu opioid receptor pharmacological variability (82). Other explanations of pharmacological variability are thought to be attributable to physical combinations of opioid receptors associating functionally within the bilipid membrane with other receptors of their class [e.g., mu-mu homodimers (83)] or other opioid receptors [e.g., mu-delta heterodimers (84)] or other non-opioid receptors [mu-alpha2A heteromers (85)] among others.

In previous eras, the opportunity to understand the opioid receptor-ligand interaction depended on the ability of the medicinal chemist to manipulate the molecule. Now, the ability to physically alter the receptor(s) themselves facilitated the resolution of the anatomical features of the receptors important for extracellular ligand binding and the aspects of the intracellular components of the receptor important for signal transduction (82). Identification of the physical receptors enabled molecular manipulations to delete or “knock-out” (KO) expression of the mu, delta, and kappa receptors in mice. In different “mu knock-out mice” generated by diverse research groups, the effects of morphine on analgesia, inhibition of GI transit, immunosuppression, hyperlocomotion, reward, withdrawal, and respiratory depression were consistently and absolutely not detected (86). In contrast, morphine's effects remained detectable in both delta KO (87) and kappa KO (88) mice. This genetic specificity solidly placed the mu opioid receptor as the primary opioid receptor that has driven the therapeutic and adverse effects of opium and opium derivatives that have concurrently benefited and plagued humanity for millennia.

The cloning of the opioid receptors also enabled precise pursuit of their expression in and trafficking throughout the peripheral and central nervous systems with the development of molecular tools such as receptor antibodies (89–91) and mRNA strategies (92). Delineating the expression of the mu opioid receptor with respect to the circuitry of the sensory nervous system ascending and descending pathways, GI tract, brainstem, ventral tegmental area, among other brain regions has been key to understanding the complex pharmacology of opioids in terms of both therapeutic and adverse effects. Tracking receptor behavior under conditions of opioid exposure has revealed differential cellular internalization of the receptors (93). For example, morphine exposure typically does not result in receptor internalization whereas the high potency mu opioid receptor peptide DAMGO and methadone do (94). The process of opioid receptor internalization is considered to be driven by the activation of beta-arrestin which leads to association of the receptor with clathrin-coated pits and endocytosis (95). Congruent with that proposal, beta-arrestin2 knock out mice have demonstrated reduced desensitization of mu opioid receptors and reduced development of opioid tolerance (but not dependence) (96). Notably, the signaling via Gi and cAMP was unaffected. The idea that mu opioid receptor agonists can differentially drive signal transduction pathways has engendered significant effort to characterize the signal transduction profile of established opioid agonists as well as the development of new “biased” agonists intended to optimize engagement of signal transduction pathways that maximize therapeutic outcomes and minimize adverse outcomes (97). TRV130 is a mu opioid receptor-selective agonist with bias for Gi signaling over *β*-arrestin-2 signaling that was developed through empirical structure activity-based lead optimization (98). Compared to morphine, TRV130 demonstrated increased analgesic potency in mice and rats, but reduced *β*-arrestin-2 recruitment, receptor internalization, colonic motility impairment (mice), and respiratory suppression (rats)(99). In 2020 the FDA approved TRV130 (Oliceridine) for intravenous use in the treatment of severe acute pain. A review of clinical findings indicates that oliceridine demonstrates comparable efficacy to morphine with reduced incidence of gastrointestinal side effect. Whether oliceridine reduces the incidence of opioid-induced respiratory depression is not clear (100). It should also be noted that enthusiasm for the biased agonism approach has been tempered by questions raised regarding recently introduced lead biased compounds (101).

It is also recognized that for most G-protein coupled receptors, the structure of their interface with their associated signal transducers under states of agonism or antagonism has yet to be fully resolved (102). As determining the molecular sequence of each opioid receptor has been critical to understanding its role in the physiology and pharmacology of opioid receptor ligand effects, it stands to reason that resolution of the mu opioid receptor interfaces with its cognate signaling transducers may be essential in designing effective mu opioid receptor-biased ligands.

In early 2012, *Nature* published four reports of resolution of the crystal structures of the three classic opioid receptors (mu (103), delta (104), kappa (105)) as well as the amino acid-sequence related (67%), but pharmacologically distinct, nociceptin/orphanin FQ receptor (106). The crystal structures were notably resolved in ligand-bound conformations, specifically associated with their respective antagonists (107). Granier and colleagues determined the crystal structure of mouse mu opioid receptor when bound to the mu opioid receptor-selective morphinan antagonist *β*-FNA (103) and the crystal structure of the delta opioid receptor when bound to the delta-opioid receptor-selective antagonist, naltrindole (104). Wu and colleagues (105) reported the crystal structure of the human kappa opioid receptor when bound to the kappa opioid receptor selective antagonist, JDTic. Thompson and colleagues (106) described the crystal structure of the human nociceptin/orphanin FQ receptor kappa opioid receptor when bound to the kappa opioid receptor selective antagonist, peptide mimetic Compound 24. Comparison of the structure of mu, delta, kappa, and N/OFQ indicate common sites within the transmembrane (TM) domains that foster receptor interactions with the functional groups of the ligand that impact efficacy. This is also known as the “message” region of the ligands. The part of the ligand that confers receptor selectivity is known as the “address” region. Common receptor sites in distinct areas of the binding pocket, with which antagonists interact via the address region, were also identified for the four crystallized receptors (107, 108). These findings represented significant advances that would greatly inform subsequent opioid therapeutic drug design.

It is important to note that the aforementioned studies captured the receptor structures while bound to their respective antagonists, which maintains the receptors in an inactive state. Follow-up studies captured the crystal structures of mu, delta, and kappa receptors in the presence of agonists, a state of activation. For example, Huang and colleagues reported a crystal structure of the active state mu opioid receptor when bound to morphinan agonist BU72 and a receptor stabilizing G protein mimetic camelid antibody fragment (nanobody 39 or Nb39) (109). This report enabled direct comparison to the crystal structure of the inactive state of the receptor when bound to morphinan antagonist *β*-FNA (103). Subtle differences were noted in the interactions of the two ligands with the extracellular surface and ligand binding pocket of the mu opioid receptor (109). Claff and colleagues (110) defined the crystal structure of human delta opioid receptor in the presence of two distinct delta opioid receptor-selective agonists, the peptide KZGCHM07 and small molecule DPI-287; this initiative uncovered key distinctions between peptide and small molecule agonist interactions with delta opioid receptor as well as important features of opioid receptor activation and delta opioid selectivity of N,N-diethylbenazemide ligands.

Che and colleagues (111) obtained a crystal structure of a human kappa opioid receptor in the active state in the presence of the agonist MP1104 and the active-state stabilizing nanobody Nb 39 as was used by Huang et al. to stabilize the active site of the mu opioid receptor (109). Che et al. observed significant conformational alterations in activated kappa opioid receptor particularly in the binding pocket. Additionally, residues in the receptor were identified that are important in enabling biased signaling. Application of the information from this report combined with structure-based computation modeling informed the design of biased ligands MP1207 and MP1208 which are G-protein biased mu and kappa opioid receptor-selective partial agonists (112).

In order to further expand understanding of how the mu opioid receptor selectively activates the Gi signaling pathway, Koehl and colleagues (113) elucidated the structure of the mu opioid receptor complexed with Gi in its active state (bound to DAMGO). Comparing the mu opioid receptor-Gi complex to prior structures of GPCRs bound to Gs provided insight as to how the mu opioid receptor selectively favors coupling with Gi/o vs. other G proteins. Expanding on that strategy, Wang and colleagues (114) obtained a crystal structure of mu opioid receptor-Gi complex bound to PZM21 and FH210, which are G protein biased mu opioid receptor agonists. Through comparing this structure to prior crystallized mu opioid receptors complexed with *β*-FNA, BU72, and DAMGO, common sites of interaction were identified as well as distinct elements thought to be associated with biased signaling. This information guided design of new PZM21 analogs with improved pharmacological profiles.

## Conclusion

We are a few years past the arrival of the global COVID-19 pandemic which has resulted in millions of virus-related deaths worldwide and has escalated both the chronic pain public health crisis and the opioid epidemic. Pandemic-related increases in chronic pain are proposed to be expected (115) and the biomedical research and medical communities are advised to be ready to respond. After centuries of pharmaceutical research, the most effective analgesic opioid compounds in our pharmacopeia remains morphine, oxycodone, and fentanyl among other strong opioids that all continue to also carry the adverse side effects that have vexed humanity for millennia. It is noteworthy that the most effective and life-saving anti-addiction medications we have are also mu opioid receptor ligands (methadone, buprenorphine, and naloxone), each with unique pharmacokinetic and pharmacological features that yield their therapeutic effects.

The origin of the modern effort in the United States to identify strategies to control pain while avoiding addiction is attributed to the establishment in 1921 by New York City's Bureau of Social Hygiene, a Committee on Drug Addictions (116). This group later invited the National Research Council to provide leadership to the mission and the Committee (later the College) on Problems of Drug Dependence (CPDD) was established. CPDD was associated with the National Academy of Sciences, National Research Council from 1929 to 1976 after which it became an independent organization fostering exchange on addiction between scientists, clinicians, industry, and government officials. The National Institute on Drug Abuse (NIDA) was established in 1974 and has led the national initiatives on drug addiction research since. NIDA has notably supported key research programs that led to identification of the opioid receptor (54) and endogenous opioid neuropeptides (117) in nervous tissue, the cloning of the mu opioid receptor (76), identification of opioid receptor heteromers (84), development of bivalent opioid ligands (118–121) and biased agonists (122) and anti-opioid vaccines (123). These and other important discoveries were supported by members of NIH Pain consortium alongside NIDA (e.g., NINDS, NCCIH, NINR, NIDCR, NCI, NIA, NICHD, NIAMS, NIMH, NEI, NIGMS, NIDDK, NIAAA, NIMHD, NIBIB, NIDCD, NCATS, NHLBI, FIC, CC) that maintain a commitment to solving the problems of addiction and pain. The *Helping to End Addiction Long-Term* (HEAL) initiative (124), is the latest and perhaps most unifying effort to seek a new analgesic that is safe and without adverse side effects.

In 1966 Dr. Phil Portoghese wrote the following: “The problem of delineating the geometry of analgesic receptors and ideally, elucidating the chemical components which comprise such entities, will challenge the best efforts of the medicinal chemist for years to come”(42). It seems that we are now in the “years to come” and may be so for a long time. The best efforts of the medicinal chemist, pharmacologist, molecular biologist, neuroanatomist, structural biologists, clinicians, and many other experts are still challenged to understand the opioid receptors and to innovate new and safer approaches to access the notably effective alleviation of severe pains offered by the opioid medications. Together we are working toward a world in which opioid addiction is no longer an epidemic and all people receive safe and effective care for their pain.
